# Systemic and Disease-Specific Risk Factors in Vascular Dementia: Diagnosis and Prevention

**DOI:** 10.3389/fnagi.2017.00333

**Published:** 2017-10-17

**Authors:** Efraim Jaul, Oded Meiron

**Affiliations:** ^1^Geriatric Skilled Nursing Department, Herzog Hospital, Hebrew University, Jerusalem, Israel; ^2^Clinical Research Center for Brain Sciences, Herzog Hospital, Hebrew University, Jerusalem, Israel

**Keywords:** prodromal stage, vascular cognitive impairment, comprehensive geriatric assessment, cerebrovascular risk factors, prevention

## Abstract

In order to prevent the onset of vascular dementia (VaD) in aging individuals, it is critical to detect clinically relevant vascular and systemic pathophysiological changes to signal the onset of its preceding prodromal stages. Identifying behavioral and neurobiological markers that are highly sensitive to VaD classification vs. other dementias is likely to assist in developing novel preventive treatment strategies that could delay the onset of disruptive psychomotor symptoms, decrease hospitalizations, and increase the quality of life in clinically-high-risk aging individuals. In light of empirical diagnostic and clinical findings associated with VaD pathophysiology, the current investigation will suggest a few clinically-validated biomarker measures of prodromal VaD cognitive impairments that are correlated with vascular symptomology, and VaD endophenotypes in non-demented aging people. In prodromal VaD individuals, distinguishing VaD from other dementias (e.g., Alzheimer's disease) could facilitate specific early preventive interventions that significantly delay more severe cognitive deterioration or indirectly suppress the onset of dementia with vascular etiology. Importantly, the authors conclude that primary prevention strategies should examine aging individuals by employing comprehensive geriatric assessment approach, taking into account their medical history, and longitudinally noting their vascular, systemic, cognitive, behavioral, and clinical functional status. Secondary prevention strategies may include monitoring chronic medication as well as promoting programs that facilitate social interaction and every-day activities.

## Introduction

Vascular dementia (VaD) is considered after Alzheimer's Disease (AD), as the most prevalent type of dementia (Lobo et al., 2000). According to the medical literature it is not clear how to distinguish between AD and VaD (Chui et al., 2000). More so, there is a lack of standardized medical evaluations and it is difficult to validate them in the clinical setting (Gorelick et al., 2011). Therefore, a few cardinal questions emerge regarding the detection and prevention of VaD. First, is VaD a distinct clinical condition? Second, does VaD induce etiological precursors to AD pathophysiology? Accordingly, in the current theoretical investigation, we will suggest that VaD could lead to increased cerebrovascular impairments (e.g., ischemia) culminating in accelerated neurodegenerative processes. Alternatively, neurodegenerative processes initiate cerebrovascular pathologies (i.e., mixed dementia). Importantly, since both types of dementia are associated with cerebrovascular and cardiovascular conditions in the aging population, we raise a third fundamental question, can non-specific dementias represent a geriatric heterogenic syndrome related to a multi-factorial clinically-high-risk state culminating in the onset of VaD and AD such as in mixed dementia?

In order to explore these questions, we hypothesized that if the underlying pathophysiology is related to vascular conditions, prevention of abnormal vascular episodes (e.g., myocardial infarction, cerebrovascular accident) as well peripheral vascular disease, and promotion of healthy life styles (weight, diet, physical activity) will reduce the prevalence of cerebrovascular chronic pathologies, leading to lower prevalence of VaD (Christmas and Anderson, 2000). Accordingly, the authors aim to shed light on vascular conditions (hypertension, hyperlipidemia, hyper-coagulation, and arrhythmia), which represent the systemic pathological state that leads to vascular dementia, unlike AD-based primary pathophysiological processes that directly lead to specific neurodegenerative cellular processes (e.g., producing abnormal levels of tau proteins; Mattsson et al., 2009). Hence, we propose that VaD represents a geriatric syndrome, which could also indicate a clinically-high-risk state that increases the onset-probability of other dementias such as AD (and possibly frontotemporal dementia). In order to support this statement, we would like to discuss a few examples as observed in the clinical setting.

## Clinical presentation of vascular dementia

In geriatric clinical evaluations, the most common method to distinguish VaD from AD requires identifying the location of the primary vascular abnormality in VaD vs. neurodegeneration (hippocampal atrophy) observed in AD, usually at subcortical or cortical regions (Staekenborg et al., 2008). The cortical regions usually include damage to the larger blood vessels leading to lateralized sensorimotor symptoms. In contrast, the subcortical regions include small vessel thrombosis and embolism leading to brain parenchyma or chronic ongoing atherosclerosis. These subcortical vascular pathological conditions may negatively impact the deeper cerebral nuclei (e.g., basal ganglia nuclei) and white matter pathways (e.g., commissural/projection fibers). The clinical manifestations of these particular subcortical-vascular conditions might be subtle, including; focal motor signs (swallowing difficulties), gait disorder with instability and recurrent falls, urinary frequency/urgency, mild delirium (transient confusion), personality, and mood changes (depression, apathy, emotional fluctuations), and non-detectable mild cognitive impairments (MCIs) associated with accelerated decline in executive attention (Meiron et al., 2017). Mood changes and accelerated cognitive decline, could initially be difficult to detect in the prodromal pre-dementia stages, however, it is critical to detect these non-salient behavioral changes in order to signal the approaching onset of recurrent falls, delirium and swallowing difficulties, which could lead to increased hospitalizations and reduced quality of life. Identifying these early risk factors (i.e., accelerated cognitive decline between age 45–65), could direct family/geriatric clinicians to apply early preventive treatment strategies that will temporarily delay the onset of psychomotor symptoms (e.g., impaired spatial orientation, recurrent falling, and immobility), resulting in increased longevity, and sustain independent everyday-functioning in elderly high-risk patients with VaD etiology. In many post-stroke patients, it is difficult to distinguish VaD onset from AD progression because the pre-stroke cognitive status was not noted. In addition, there are often sub-clinical-undetectable white matter lesions in post-stroke dementia patients. To increase the sensitivity for detecting and treating these pre-dementia conditions in pre or post-stroke patients, we recommend routine assessment of vascular and systemic risk factors to reduce susceptibility of VaD onset.

The main challenge is in detecting these geriatric clinical indications (Staekenborg et al., 2010). Unfortunately, many of the patients are usually referred to multiple specialized physicians (e.g., urologist, psychiatrist, neurologist) in response to the patients presenting symptoms, leading them to focus only on one component of the disease (depression, gait, instability, and urinary incontinence) instead of examining the entire physiological condition (medical history, full organs examination, blood test, vascular/brain imaging). In addition, geriatric vascular symptoms fluctuate according to the patient's daily activities/periods, and the effects of chronic drug treatment inadvertently lead clinicians toward misdiagnoses, which are likely to result in ineffective treatments. For example, due to the patient's bladder urgency the physician could prescribe antispasmodic drugs with anticholinergic action. However, the adverse effects of these drugs could be bladder retention and cognitive impairments which could deteriorate the patient's condition. If the same patient has other systemic risk factors that increase the frequency of recurrent falls, delirium and urinary infections the risk for development of VaD in later years significantly rises.

## Systemic and specific risk factors

The main risk factors for VaD can be delineated by three prodromal or ongoing pathophysiological states; (1) Aging processes in the vessels (2) Cardio-vascular risk factors (3) cerebrovascular risk factors. Aging processes in vessels increases the risk for systemic pathophysiological processes (arteriosclerosis), and the risk for cerebrovascular damage. In relevance, atheroma deposits on the intimae of the arteries occlude the diameter of the lumen or disturb the blood flow. The thickness of the intima (due to proliferation of smooth muscle cells and connective tissue), leads to rigid and tortuous vessels. Pathological aging processes and cardio-vascular risk factors are also expressed by metabolic syndrome conditions; obesity (BMI 30 kg/m^2^ or more), hypertension, insulin resistance, dyslipidemia, and increased waist circumference. One study reported that elderly people with cardiovaskular risk factor had hazard risk (HR) of 3.7 for developing dementia during the observational period (Solfrizzi et al., 2010). Another research found that MCI or Vascular Cognitive Impairments (VCI) are highly associated with VaD onset (Segura et al., 2009). Vascular brain damage due to increase cardiovascular risk factors can be characterized by two categories; clinical stroke (damage of large arteries) or subclinical “lacunar” or “silent” brain infraction (injury of small arteries). After clinically manifested stroke, dementia or cognitive impairment develops in 6–32% patients during the follow up period of 3 months to 20 years (Ivan et al., 2004; Rist et al., 2013). In a cohort-study of patients after lacunar stroke, related to small vessels diseases, 50% of the patients suffered later from MCI or dementia (Jacova et al., 2012). In a systematic review and meta-analysis of 73 papers published between the years of 1950 and 2009 examining pre-stroke and post-stroke dementia patients, the rate of dementia onset ranged between 7.4 and 41.3% (Pendlebury et al., 2009). After adjustment of the methods and mix cases, findings indicated that 10% of the patients with first stroke had pre-stroke dementia, and 10% of the patients subsequently developed dementia, while more than 33% of the patients developed dementia after a recurrent stroke (Pendlebury et al., 2009). These finding emphasizes the important role of secondary prevention (e.g., statin prescription for lowering cholesterol and triglycerides, antiplatelet drug and anticoagulant use in order to prevent thromboembolism), immediately after the first stroke. Systemic factors such as high serum levels of homocysteine or LDL-lipoproteins, hypercoagulability conditions, diabetes mellitus with inadequate glycemia control, smoking, and obesity should be examined and treated within primary and secondary prevention strategies.

## Early detection and prevention of vascular dementia

In attempt to support our theoretical premise it is prudent to further examine our theoretical model by asking “why is it clinically relevant to distinguish VaD from other dementias?” The main clinical benefit in identifying non-demented prodromal VaD cases vs. non-demented prodromal AD cases (such as detecting subtle age-related vascular vulnerabilities by utilizing known disease-specific endophenotypes), could lead to early preventive interventions in a critical time point preceding the pathophysiological phenotypical expression of VaD. Endophenotypes are physiological markers (e.g., hippocampal atrophy in individuals with Alzheimer's disease) in humans that are genetically correlated with disease liability (Glahn et al., 2007). Endophenotypes are disease-specific biomarkers that are generally closer to the action of the gene, and thus exhibit higher genetic-risk sensitivity for specific neurological disorders (Glahn et al., 2007). Suppose, for example, that a specific VaD endophenotype is discovered and that the association of that endophenotype with white matter lesions observed in VaD prior to phenotypical expression (such as mild cognitive impairment ordelirium) is proved. The finding could pawed the way for subsequent researches for novel medication which modulate that endophenotype. In the future, evidence-based treatment strategies which prevent cerebral ischemic episodes as well as the consequential onset of dementia could be finally established. Some researchers proposed the abnormal expression of myelin basic protein (MBP) to be the endophenotype for development of VaD but further validation studies were needed before definite proof of this concept (Bjerke et al., 2011; Meiron et al., 2017). Other research groups favored the assessment of total tau protein as a specific marker for AD. It has been observed that total tau protein levels in the CSF were significantly increased in AD vs. both VaD and healthy controls (Bjerke et al., 2011). In conclusion, the markers of prodromal subtle-phase that precedes VaD promised an era of emerging preventive and therapeutic strategies which could suppress or delay the onset of debilitating dementia cognitive-symptoms, as well as altering neurodegenerative disease progression.

On a cognitive level, high-risk elderly pre-dementia individuals may exhibit VCI (O'Brien et al., 2003), or non-demented MCI, or non-salient accelerated cognitive decline (pre-MCI state that is less severe in comparison to amnesic or progressive MCI), therefore it is important to detect significantly lower verbal memory scores in MCI vs. non-MCI controls using standard cognitive testing measures such as the Rey Auditory Verbal Learning Test (RAVLT) and the California Verbal Learning Test (Deiber et al., 2009; Albert et al., 2011). In most cases, verbal memory is the most prominent cognitive-domain that gradually deteriorates and this pre-condition is likely to progress to AD in 12% of MCI patients within a consecutive 24-month period or between 40 and 60% cumulatively, over a life-time period (Hua et al., 2008; Babiloni et al., 2010). However, in prodromal VaD, the impact of MCI symptoms on daily activities is less apparent (particularly to the family and immediate community) than subtle gait impairments leading to increased risk of falling (Jokinen et al., 2006). Therefore, it is clinically essential to identify early high-risk MCI cases with vascular history before they exhibit significant cognitive impairments that lead to an irreversible physical condition due to spatial disorientation or falling. In support, early detection of increased dual-task (e.g., walking while performing a cognitive task) deficits in elderly controls (Manor et al., 2016) could be perceived as an early VaD phenotypical expression preceding gait problems and recurrent falls, particularly if the person has cardiovascular history. As these types of dual-task assessments are associated with left prefrontal cortex excitability as well as with executive attention tasks (Meiron et al., 2013) that are associated with prefrontal-cortex functional connectivity (Meiron and Lavidor, 2014), we can employ these “prefrontal activity” neuro-cognitive testing procedures to detect accelerated pathological cognitive decline. In support, progressive MCI patients display significantly reduced prefrontal theta EEG activity (during the onset of a working memory trial requiring the correct rejection of a non-target stimulus) vs. stable MCI, and vs. healthy controls. Hence, if an elderly person without any memory complaints begins to show clinically significant deterioration in dual-task scores (Manor et al., 2016), as well as attaining a verbal memory score (e.g., Rey Auditory Verbal Learning Test) that is at least 1.5 *SD* lower than the age-matched group-mean (Albert et al., 2011), we suggest that it would be advisable to proceed with functional EEG imaging to exclude or identify progressive MCI (Deiber et al., 2009). Additionally, clinical assessment should include the Petersen MCI criteria, wherein single domain amnestic MCI will be seen more as a predictor for AD and/multi-domain MCI may be more indicative of underlying vascular pathology (Albert et al., 2011). Finally, in order to rule out VaD diagnosis, we suggest that white matter lesions should be examined (associated with ineffective cerebral connectivity) along with hippocampal-atrophy, and both conditions should be excluded (Hua et al., 2008; Cao et al., 2010; Ardekani et al., 2014). All these procedures (MRI, EEG, Hachinski Ischemic/Ischemia Scores/Scale) are available and are non-invasive, facilitating the cooperation of more prospective elderly patients willing to be examined and clarifying the clinical picture for the treating physicians. In support, the Hachinski Ischemic Scores can be applied routinely to reliably distinguish vascular dementia from other dementias (Moroney et al., 1997).

## Discussion

In response to the diagnostic and clinical implications for early detection of prodromal VaD, our theoretical perspective suggests that VaD represents a final pathway induced by a multi factorial pathophysiological (e.g., risk factors such as lacunar stroke and atherosclerosis) state leading to a geriatric syndrome. Primary VaD prevention strategies should focus on modifying daily life styles such as: smoking, caloric intake, carbohydrates and salt in diet, aerobic and anaerobic physical exercise. Secondary prevention strategies may include monitoring of antilipemics, antidiabetics, antihypertensives, antiplatelets and anticoagulants medications, and tertiary prevention measures such as rehabilitation programs after stroke as well as promoting programs that facilitate social interaction and every day independent activities are also important.

In elderly person, we have to allow sufficient blood and oxygen perfusion to, and in the brain, hence, it is advisable to be cautious with aggressive hypo-perfusion medications, which lower blood pressure and glucose levels in the brain. To accomplish an effective holistic approach in preventing or treating MCI/VaD it is critical that the family physician coordinates the treatment with other expert-clinicians (e.g., social worker, occupational therapist, neuropsychologist) in order to advance a multi-disciplinary prevention-treatment to delay the onset of dementia and related functional impairments.

Particularly in its prodromal early stages, undiagnosed VaD progression in geriatric populations stems from the lack of disease-specific criteria to identify VaD as a distinct clinical entity. Does this imply a clinical need for an appropriate new diagnostic entity associated more with VCI (O'Brien et al., 2003). VCI can result from multiple pathophysiological pathways. For instance, post-stroke patients display focal cognitive deficits related to the area of infraction or/and suffer from hypo-perfusion affecting deeper neural structures (associated with global cognitive impairments) indicating a systemic cerebrovascular atherosclerosis. VCI can be detected in patients suffering from a manifested and subclinical stroke, silent white matter lesions and arrhythmias (e.g., atrial fibrillation). Systemic chronic diseases significantly promote the pathogenesis of blood-vessel diseases. Sporadic VCI evolution to MCI (i.e., mild neurocognitive disorder; American Psychiatric Association, 2013) may represent a shift from mild cognitive symptoms to a deterioration of functional capacity status (e.g., ADL and cognitive impairments) that is diagnosed as mixed dementia, associated with AD and VaD debilitating cognitive impairments.

## Conclusion

The authors suggest that the optimal prevention-treatment approaches to reduce VaD prevalence or severity require early *comprehensive geriatric assessment*. The term “comprehensive” indicates taking into account at the overall clinical features and not just the presenting symptoms (gait, urinary urgency, depression). For instance, Hachinski Ischemic Scores (HIS ≥ 7) are considered a sensitive and specific diagnostic marker for identifying VaD with high inter-rater reliability (Moroney et al., 1997). Thus, HIS scores in combination with neuroimaging, as well as vascular pathology imaging (e.g., pulsed arterial spin labeling, pASL; Cantin et al., 2011) could reliably indicate VaD pathophysiology. To accomplish an effective multi-factorial approach in identifying or preventing VIC/VaD onset it is critical to apply early multidisciplinary prevention-treatments utilizing systemic-vascular-neurocognitive quantifiable cross-validated markers to delay or possibly prevent the onset of dementia and related functional impairments.

## Author contributions

Both authors contributed equally to the conception and composition of this paper.

### Conflict of interest statement

The authors declare that the research was conducted in the absence of any commercial or financial relationships that could be construed as a potential conflict of interest. The reviewer ND and handling Editor declared their shared affiliation, and the handling Editor states that the process nevertheless met the standards of a fair and objective review.
